# Long Exhalations as Complementary Treatment for Chronic Pain: A Pilot Study

**DOI:** 10.3390/jcm14227975

**Published:** 2025-11-11

**Authors:** Jorge Castejón-España, Sven Vanneste

**Affiliations:** 1 Lab for Clinical and Integrative Neuroscience, Trinity College Institute for Neuroscience, School of Psychology, Trinity College Dublin, D02 R123 Dublin, Ireland; castejnj@tcd.ie; 2 CompassPhysio, A83 YW96 Enfield, Ireland; 3 Global Brain Health Institute, Trinity College Dublin, D02 R123 Dublin, Ireland; 4 Brain Research Centre for Advanced, International, Innovative and Interdisciplinary Neuromodulation, 9700 Ghent, Belgium

**Keywords:** chronic pain, breathing, long exhalations, physiotherapy

## Abstract

**Background**: Chronic pain (CP) is one of the biggest burdens for health systems across the globe. It frequently presents in conjunction with comorbidities and considerable challenges for the maintenance of homeostasis and well-being. The lack of long-term effective treatments requires further attention and innovative approaches from the health care community. The present observational study aims to prove the feasibility of a breathing protocol focused on long exhalations (LEx) as a complementary treatment for CP populations. **Methods**: Eighteen CP patients (nine men and nine women) were selected for this observational pilot study. The inclusion criteria were having pain for more than 3 months, not having any previous experience with breathing exercises and not having a clinical diagnosis for the condition suffered. In addition to the usual physiotherapy care, the participants were trained in breathing techniques and the effects of LEx. Before each appointment, the Numeric Pain Rating Scale (NPRS), Pain Catastrophising Scale (PCS) and exhalatory times were registered. The data analysis consisted of a repeated measures ANOVA and a Pearson Correlation Coefficient. **Results**: A total of 18 participants completed the breath intervention and the assessments. All participants improved their exhalation times by 4.78 s (SD = 3.19) or 64% (F = 45.62, *p* < 0.001) and their pain scores: NPRS by 2.55 units (SD = 2.2) or 47% (F = 34.19, *p* < 0.001); and PCS by 11.34 units (SD = 16.05) or 33% (F = 24.05, *p* < 0.001). There was a moderate positive correlation (r = 0.49, *p* = 0.05) between exhalation times and NPRS. **Conclusions**: Breathing techniques focused on LEx in combination with the usual physiotherapy care are a feasible pain management protocol to reduce subjective pain perception and pain catastrophizing scores. Long-term studies with bigger samples might benefit from the inclusion of accurate and reproducible measures for exhalation times.

## 1. Introduction

Chronic pain (CP) is defined as any pain that persists for more than three months [1,2]. It has a prevalence of 20% across the globe and an estimated direct and indirect cost of USD 560–650 billion per year only in the US [3]. Generally, the cost per person per year ranges between USD 6400 to USD 9231 [4,5,6]. Such elevated costs not only emanate from its palliative treatment but also from its frequent comorbidities: anxiety, depression, sleeping disorders, etc. [7].

CP is a condition that goes beyond the endogenous physiological restoration of the body, and is the result of the interaction of three independent anatomical pathways: the lateral or painful pathway, the medial or suffering pathway and the inhibitory or pain suppression pathway [1,8]. Suffering is a common feature among the frequent cognitive CP comorbidities and, if sustained over time, it might lead to increased stress and allostatic load [9,10].

Stress is a physiological state in response to an internal or external threat that is both a cause and consequence of a variety of psychiatric conditions [11,12]. Its main hub is the Locus Coeruleus (LC), a small cluster of neurons and glial cells located in the posterior aspect of the pons that is the sole source of norepinephrine for the cerebral cortex [13,14] The LC is a neuro-modulatory system in which a small group of neurons regulate arousal, attention, pain modulation, sensorimotor plasticity, breath control and many other functions [14,15,16,17,18,19].

The mere existence of a hub with such a wide scope of action opens the possibility of designing novel treatments for managing CP and its different comorbidities. Previous research has proved that LC is sensitive to CO_2_ changes and that it has strong neuroanatomical connections with breathing pattern generators [15,20,21]. Hence, breath control might be proposed as a potential CP modulator via LC activity. 

Breathing techniques have been used for millennia in social and religious contexts, and they have proven benefits for anxiety, depression, memory consolidation and blood pressure, among others [22,23]. To date, the most studied breathing techniques have been diaphragmatic or abdominal breathing and resonance breathing. However, little is known about breathing techniques and their potential implications across different body systems (musculoskeletal, neurological, respiratory, etc.). In that direction, long exhalations (LEx) have been proven to stimulate parasympathetic activation and are a simple and reproducible method of lengthening the diaphragm and creating a reflex activation of the abdominal muscles [24,25,26]. Hence, LEx are positioned as a promising clinical tool for modulating stress-related changes in CP.

In this pilot study, we explore the feasibility of a breathing protocol based on LEx as a complementary treatment for patients with CP. If the feasibility outcomes are achieved, future research should investigate the long-term effects of this breathing programme in larger populations with a control group.

## 2. Methods

### 2.1. Participants

Eighteen CP subjects took part in this pilot study (mean age 46.44 ± 12.14 years; nine males, nine females) between September and December 2022. The inclusion criteria included (i) having suffered pain in that area for at least 3 months, (ii) not having previous experience with breathing techniques and (iii) not having an existing assessment at the clinic for their current condition. The 18 CP subjects were classified as chronic secondary musculoskeletal pain (n = 12), and chronic post-traumatic pain (n = 6) based on the International Association for the Study of Pain diagnostic guidelines [2]. Table 1 also includes details of the suspected pain mechanisms based on clinical assessment. The Ethics Committee of Trinity College Dublin approved to retrospectively analyse the data (TCD20-11). All patients signed an informed consent as part of their consent to receive treatment at the clinic.

### 2.2. Protocol Before Each Session

Participants were retrospectively selected from the caseload of a private physiotherapy clinic, where they were presented with an initial form to be completed prior to entering the treating room. The form included a Numeric Pain Rating Scale (NPRS), a 13-item Pain Catastrophizing Scale (PCS) and a timed maximum exhalation (in seconds) after an inspiration of 4 s [27]. This protocol was applied during the month of January 2024 as part of an improvement of clinical practices in the service. 

The NPRS contained 11 scale ranges from 0 to 10 (0—no pain and 10—worst pain possible). Participants were instructed to mark the number that better represented their pain on the day of the assessment. This scale has been validated for assessing pain experience and intensity [28]. It has a high intraclass correlation (r = 0.95) and shows consistency with other subjective pain scales such as the Visual Analogic Scale [29]. NPRS has shown higher reliability for rheumatic and CP patients than other subjective scales [30]. 

The 13-item PCS measures the context of the painful experience and contains three subcomponents: Helplessness, Rumination and Magnification [27]. It has shown an excellent internal consistency for the total score (α = 0.87–0.93) and adequate to excellent for its subcomponents: Rumination (α = 0.85–0.91), Magnification (α = 0.66–0.75) and Helplessness (α = 0.78–0.87) [27,31].

The selected participants completed the second form on their follow-up appointment. That second consultation was always booked with the same therapist and within a fortnight of the first one.

### 2.3. Session

The clinical assessment and treatment were prescribed by a physiotherapist with 10 years of experience following the recommended protocols for each specific case. These treatments always included pain education, specific exercise prescriptions and hands-on techniques if required. Complementarily, each patient was asked to perform a long exhalation and received feedback and education about breath control, anxiety and its role in facilitating pain and suffering. They were also trained to control their exhalations and instructed to aim to improve the duration of their exhalation beyond 15 s. The participants received a complete exercise plan through Physiapp^®^ (accessed in 2022) with a specific exercise named “Long exhalations” that reminded them to perform LEx daily. There was no specific prescription for the frequency or how long to practice their breath training.

On the second appointment, the therapist measured a second exhalation and, in case of lack of progress, the participants were encouraged to share impressions about the breathing techniques and ask questions about them. Subjects that were improving were motivated to continue with the same frequency and dynamic of breath training.

### 2.4. Objectives

#### 2.4.1. The Scientific Objective of This Study Is

Assess the feasibility and potential effectiveness of breathing exercises focused on LEx in reducing suffering and pain perception among the CP population.

We hypothesise that suffering (PCS) and subjective pain perception (NPRS) can be reduced through breath training focused on LEx.

#### 2.4.2. The Feasibility Objectives of This Study Are

Determine whether the selected outcome measures (PCS, NPRS and exhalation times) are appropriate for detecting breathing-related changes over the course of the study.

Evaluate the viability of using the selected outcome measures to track objective measurements of pain perception and breathing over time.

### 2.5. Outcomes

The primary outcome of the study is to observe improvements beyond the Minimal Clinically Important Difference (MCID) on the NPRS before and after intervention.

#### The Secondary Outcomes of the Study Are

Evaluate the impact of LEx on patients’ cognitive and emotional responses to pain, as measured by changes in PCS scores.

Track the improvement in patients’ ability to perform LEx, indicating adherence to and effectiveness of the breathing technique training.

### 2.6. Feasibility Criteria

The feasibility criteria are based on the MCID, that are a 2-point reduction for the NPRS and an overall score reduction of 33% for PCS. In the case of the duration of the exhalations, the feasibility criteria are an increase in the duration of the exhalation in seconds.

### 2.7. Data Analysis

Once the data were collected, it was processed with SPSS Statistics (v29.0). A repeated measures ANOVA was conducted with pre- and post-session assessment for the NPRS, the subscale of the PCS and the total score as a within-subjects variable. Furthermore, a Pearson Correlation was applied to examine the correlation between the breath (difference between session 1 and session 2) and the pain (difference between session 1 and session 2) scores.

## 3. Results

**Figure 1:**
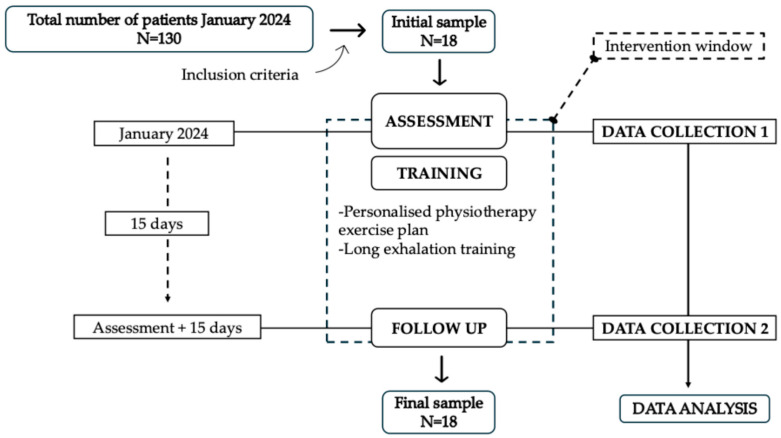
Participant flow and intervention window.

This pilot study analysed the effects of breath training on exhalation rates and self-reported pain scores (NPRS and PCS) in a CP sample. Figure 1 depicts the participants’ flow through the time window utilised in this study. Each of the eighteen participants completed two clinical assessments and 15 days of long exhalation training in addition to an individualised exercise plan. A total of 18 participants completed the breath training and the pre- and post-intervention assessments. All participants indicated that the breathing control exercise was feasible.

### Pre- and Post-Intervention Values

Post-intervention, there was a significant increase in the mean value of exhalation times by 4.78 s (SD = 3.19), equivalent to a 64% increase (see Table 2). In addition, pain scores were significantly reduced by 2.55 units (SD = 2.2) for the NPRS, and 11.34 units (SD = 16.05) for the PCS—equivalent to 47% and 33%, respectively. Furthermore, the three different subcomponents of the PCS showed a significant decrease after intervention (Rumination 4.44 units (SD = 3.39), Magnification 2.83 units (SD = 3.34) and Helplessness 3.22 (SD = 4.68)). See Table 2.

To further investigate the association between the breathing intervention and subjective pain scores, Pearson Correlations were calculated. The results showed a significant correlation between breath and NPRS, indicating that an increase in the length of exhalation times is associated with lower NPRS scores. No significant correlation was obtained between breath and the subscales or the total score of the PCS, or between the PRNS and PCS. However, a significant correlation was obtained between the subscales of the PCS and the total score of the PCS. See Table 3.

## 4. Discussion

This pilot study has evaluated the feasibility and efficacy of daily slow breath training focused on LEx on the perception of pain among eighteen CP patients. In contrast with previous research, this pilot has not attempted to assess an immediate reaction to a specific breathing technique. Instead, it has analysed the consequences of steady and progressive adaptations to LEx. We observed a significant decrease in both NPRS and PCS, accompanied by an increase in exhalation times. These results point towards a potential effect of this type of breath training in modulating CP perception. In this section, we will explain the reasons behind the selection of this breathing technique. Subsequently, we will explore the possible explanations for the observed changes alongside potential downstream biomechanical, cardiorespiratory, and neurological changes that could arise in CP patients from this intervention. This section will then conclude with limitations and changes to consider for future implementations of the suggested protocol.

### 4.1. Interpretation of Findings

The eighteen participants of this study showed a short-term decrease in their NPRS and PCS scores while increasing exhalation times. The average reduction for the NPRS is greater than 1.65 MCID for general pain and 2.00 points or 33% for CP [28,32]. However, it is important to note that the NPRS are often self-reported as non-clinically meaningful for CP patients [33,34,35] (Table 2).

There were significant differences in the subsections of the PCS (Rumination, Magnification, Helplessness), and the overall score reduction of 33% is under the MCID (38–44%) [36]. However, if we look at the total score of the scale, the number of patients with a total score higher than 30 (MCID for catastrophizing) decreases from 10 to 4 (60% reduction). This might suggest a powerful effect of this protocol among the pain-catastrophising population. The catastrophising reduction effect is quantitatively similar to previous research but much quicker and more efficient than other interventions [27,37,38]. No statistical correlation was found between breath exhalation rates and PCS. 

Additionally, we observed a significant positive correlation between the increase in breathing times and the reduction in NPRS scores, but not with PCS or its subcomponents. This, together with the overall PCS reduction of 33%, points towards a direct effect of breathing on subjective pain perception and a more intricate effect on suffering. Interestingly, similar modest effects in the reduction in PCS have been observed with physical, psychological and pharmacological therapies [39]. A bigger sample size, the addition of subsequent follow-ups and the refinement of the breathing time measurement tool might offer answers to the different effects observed in this pilot.

### 4.2. CP Is Not Acute Pain

Previous research has explored the effects of breathing techniques on immediate changes in acute pain perception. Different conclusions have arisen, but there is a general lack of consensus about the interpretation of the obtained results and their explanation [40]. The definition of CP as pain that persists for more than 3 months is explained by the time it takes for the complex variety of neuroplastic changes to spread through the peripheral and central nervous systems. These adaptations affect both nociceptive and perceptive pathways and are frequently interlinked with previous experiences and traumas [41]. 

Neurophysiologically, exposure to acute pain is likely to generate stress-like autonomic responses (blood pressure, heartbeat, breath frequency, catabolic metabolism, etc.) to cope with the triggers of the nociceptive input [42,43]. However, if perpetuated in time, the constant exposure to nociceptive inputs will expose the autonomic nervous system to an increased necessity to adapt, which can also be termed as allostatic load [9,44]. In brief, CP is distinct from acute pain in that CP patients bear an increased allostatic load that represents an unsuccessful attempt of the system to adapt to a persistent nociceptive threat. 

### 4.3. Clinical Implications

This pilot study presents an innovative and holistic approach for the management of pain perception in CP populations. The short-term improvements observed in NPRS and PCS scores, in addition to the increase in exhalation times, suggest a potential effect of this type of breath training on CP perception. 

**Figure 2:**
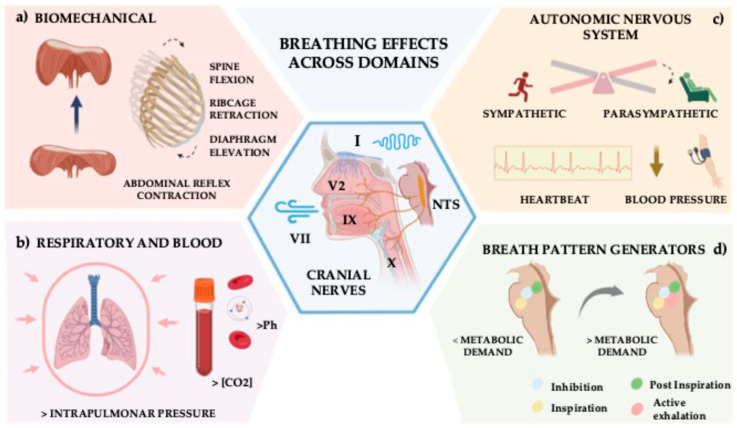
This figure depicts the effects of breath across different systems in the human body. The centre of the figure represents the effects on the cranial nerves (olfactory, trigeminal, facial, glossopharyngeal and vagus). Any given breathing frequency will modulate brain activity through the rhythmic inputs collected by these nerves. (**a**) Biomechanical changes will occur at the ribcage, diaphragmatic and spinal levels, the main source of these changes being the lengthening (elevation) of the central tendon of the diaphragm, (**b**) increase in intrapulmonary pressure due to the retraction of the ribcage or the opening of the mouth, together with an elevation of Ph and CO_2_ concentration in the blood, (**c**) shift towards the parasympathetic autonomic nervous system coupled with a decrease in blood pressure and heartbeat with increasing Heart Rate Variability, (**d**) Changes in the central neurologic control of the breath from a low metabolic state to a high-demand metabolic state. The isolation of any of these changes does not provide enough ground for the observed pain reduction. We believe that the temporo-spatial summation of these neurophysiological changes is the foundation of the observed effects. NTS: Nucleus Tractus Solitarius. The figure was created using PowerPoint and BioRender.com (BioRender, 2023).

The ability to perform longer exhalations implies a volitional modification of the breathing pattern that combines (i) the controlled lengthening of the inspiratory musculature (mainly external intercostals, diaphragm and scalenus muscles), (ii) the volitional activation of the exhalation muscles (mainly abdominals and internal intercostals), (iii) the control of the flux of air through the upper respiratory tract (larynx, pharynx, mouth and nasal cavity), and (iv) the maximum relaxation of pleural and pulmonary stretch receptors [45,46]. All these different biomechanical and physiological changes are equivalent to the direct neurological activation of five different cranial nerves (I, V, VII, IX and X) and the somatosensory cortical areas where the trunk, mouth and pharynx are represented [47], Figure 2.

Interestingly, when all these neurological inputs reach the brain, they can modulate specific brain networks such as the salience network, whose main hub is the anterior insula and the anterior cingulate cortex [48,49]. The insula is a well-known centre for the integration of interoceptive signals (nociceptive and non-nociceptive) with inputs from the environment [50,51,52].

Additionally, the pattern of respiration implemented in this pilot has a frequency within the range of infra-slow neural oscillations (ISO) (0.01–0.1 Hz or less than 6 breaths per minute). These very slow oscillations are more prominent during sleeping and resting states and have been correlated with arousal states [53,54]. The powerful effects of these ISO might be related to their ability to influence different cognitive functions by modulating the higher-frequency neural oscillations (alpha, beta, gamma, theta, delta) that are nested in them [55].

On this basis, a potential explanation for the observed pain modulatory effects could be the interaction of multidomain inputs within the central nervous system. However, this is an area that requires more attention and coordination between multiple disciplines, including neuroscience, psychology, physiotherapy and even sports science.

## 5. Limitations and Insights for Future Research

For interpreting these findings, readers and future researchers should consider certain limitations. Firstly, the reduced sample size, the 2-week follow-up, the lack of a control group and the non-blinded design of this pilot reduce the applicability of the findings and greatly increase the risk of bias. Secondly, when measuring the LEx, we identified the necessity of (i) using a spirometer to measure the exhalation times with an appropriate level of accuracy and (ii) standardising a protocol for each participant that includes a specific number of respirations, position and education according to the existing health literacy. Thirdly, another source of confounding factors might be the type of physiotherapeutic treatment applied alongside the breath training, and the fact that the data were collected from a private physiotherapy company where patients pay a fee per session. Finally, given the small sample size and the nature of the clinical environment where the data were collected, there are specific subtypes of CP that were not covered in this pilot (cancer, visceral, neuropathic, etc.).

## 6. Conclusions

In this retrospective observational pilot, we observed that a 2-week breath training protocol focused on Lex, in addition to the usual physiotherapy care, has produced an increase in exhalation times and a decrease in both NPRS and PCS. Breath training focused on Lex seems to have a direct effect on NPRS reduction and an indirect effect on PCS scores. This suggests that incorporating breathing techniques focused on LEx into usual physiotherapy care for CP patients may favourably modulate pain experience and overall suffering. Future research should delve into this cost-effective multidomain type of breathing as a potential tool to further improve pain management strategies for CP populations.

## Figures and Tables

**Table 1:** Participants’ distribution across pain types and mechanisms.

Pain Type	Pain Mechanism			
	Nociceptive	Nociplastic	Neuropathic	Total
Chronic secondary musculoskeletal	6	4	1	12
Chronic post-traumatic	4	2	2	6

**Table 2:** Repeated measures ANOVA for pre- and post-measurements for each of the studied variables.

	Pre	Post	Difference Pre-Post (Percentage)	*F*	*p*
***Breath (s)***	7.44 ± 2.73	12.22 ± 1.67	4.78 ± 3.01 (64%)	45.62	<0.001
NPRS	5.44 ± 1.62	2.89 ± 1.53	−2.56 ± 1.47 (−47%)	34.19	<0.001
*Pain Catastrophising Scale*					
Rumination	11.39 ± 4.62	6.94 ± 2.96	−4.44 ± 3.40 (−39%)	30.77	<0.001
Magnification	8.56 ± 3.96	5.72 ± 2.68	−2.83 ± 3.35 (−33%)	12.90	0.002
Helplessness	14.11 ± 5.86	10.06 ± 4.37	−3.22 ± 4.68 (−23%)	18.31	<0.001
Total	34.06 ± 13.07	22.72 ± 9.34	−11.33 ± 9.80 (−33%)	24.05	<0.001

Notes: Data is presented as Mean ± SD. Breath was measured in seconds. NPRS: Numeric Pain Rating Scale.

**Table 3:** Pearson correlation test for the Breath and other variables.

	NPRS	Total	Helplessness	Magnification	Rumination
**Breath**	0.49 *	0.20	0.07	0.26	0.09
**Rumination**	0.20	0.91 **	0.70 ***	0.68 **	
**Magnification**	0.25	0.83 ***	0.62 ***		
**Helplessness**	0.17	0.90 **			
**Total**	0.17				

* *p* < 0.05, ** *p* < 0.01, *** *p* < 0.001, NPRS: Numeric Pain Rating Scale.

## Data Availability

Due to privacy and ethical restrictions, the data are not publicly available but may be obtained from the corresponding author upon reasonable request and with permission of the Ethics Committee of Trinity College Dublin.
